# Muscle Shear Elastic Modulus Provides an Indication of the Protection Conferred by the Repeated Bout Effect

**DOI:** 10.3389/fphys.2022.877485

**Published:** 2022-04-29

**Authors:** Emeric Chalchat, Julien Siracusa, Cyprien Bourrilhon, Keyne Charlot, Vincent Martin, Sebastian Garcia-Vicencio

**Affiliations:** ^1^ Institut de Recherche Biomédicale des Armées, Unité de Physiologie des Exercices et Activités en Conditions Extrêmes, Département Environnements Opérationnels, Bretigny-Sur-Orge, France; ^2^ AME2P, Université Clermont Auvergne, Clermont-Ferrand, France; ^3^ LBEPS, Univ Evry, IRBA, Université Paris Saclay, Evry, France; ^4^ Institut Universitaire de France (IUF), Paris, France

**Keywords:** eccentric exercise, neuromuscular function, shear wave elasotography, muscle stiffness, downhill walking, elasticity, muscle adaptation, ultrasound

## Abstract

**Background:** The neuromuscular system is able to quickly adapt to exercise-induced muscle damage (EIMD), such that it is less affected by subsequent damaging exercise, a phenomenon known as the repeated bout effect (RBE). The objective was to determine whether the mechanical properties of the quadriceps, as evaluated by shear wave elastography (SWE), were less affected when a second bout of eccentric-biased exercise was performed 2 weeks later. It was hypothesized that the first bout would confer protection against extensive muscle damage through an adaptation of the muscle stiffness before the second bout (i.e., higher muscle stiffness).

**Methods:** Sixteen males performed two identical bouts of downhill walking separated by 2 weeks (45 min at 4.5 km.h^−1^; gradient: 25%; load: 30% of the body mass). *Rectus femoris* (RF) and *vastus lateralis* (VL) resting shear elastic modulus (µ) and EIMD symptoms were measured before and up to 7 days following the exercise bouts. Changes in neuromuscular function was evaluated by maximal voluntary contraction torque, voluntary activation level, evoked mechanical response to single and double (10 and 100 Hz doublets) electrical stimulation. An index of protection (IP) was calculated for EIMD symptoms to assess magnitude the RBE.

**Results:** EIMD symptoms were less affected after the second than the first exercise bout. RF and VL-µ increased (*p* < 0.001) only after the first exercise. RF µ was elevated up to 2 weeks after the end of the first exercise (*p* < 0.001) whereas VL µ was only increased up to 24 h. The increase in µ observed 2 weeks after the end of the first exercise was correlated with the IP; i.e., attenuation of alterations in muscle µ, 10 Hz-doublet amplitude and rate of torque development after the second exercise bout (*p* < 0.05).

**Conclusion:** We showed that muscle µ assessed by SWE was sensitive to the RBE, with a differential effect between VL and RF. The persistent increase in µ was associated with the attenuation of neuromuscular impairments observed after the second bout, suggesting that the increased muscle stiffness could be a “protective” adaptation making muscles more resistant to the mechanical strain associated to eccentric contractions.

## Introduction

One of the major feature of the neuromuscular (NM) system is its degree of plasticity in response to endogenous and exogenous influences, such as changes in loading regimes (Narici, 1999). It can translate into adjustments of structural, neural, and functional properties (Kawakami et al., 1993; Škarabot et al., 2021) of the NM system. For instance, the high or repeated mechanical stress exerted by eccentric (lengthening) exercise (ECC) on the muscle-tendon complex leads to exercise-induced muscle damage (EIMD). EIMD results in a defined set of symptoms (e.g., force loss, muscle soreness, stiffness, decrease joint range of motion, increased circulating muscle proteins) consecutive to the disruption of intracellular muscle structure, sarcolemma and extracellular matrix in the days following exercise (Fridén et al., 1984; Proske and Morgan, 2001; Paulsen et al., 2012). However, the NM system is able to quickly adapt to EIMD, such that the NM system is less affected by subsequent damaging exercise. This phenomenon is widely known as the repeated bout effect (RBE). The occurrence of RBE relies on various mechanisms including cellular, neural, inflammatory, mechanical adaptations, and extracellular matrix remodeling that might work independently or synergistically to generate muscle protection against EIMD (e.g., reduced symptoms of EIMD and enhanced recovery) when a second bout of ECC is performed (Hyldahl et al., 2017).

Previous studies suggested that neural adaptations may occur during the second bout of ECC (Duclay et al., 2008; Dartnall et al., 2011; Howatson et al., 2011). Specifically, the central nervous system may adjust its recruitment strategies to distribute the mechanical stress over a greater motor unit pool to protect the muscle from damage. While several studies showed an increased supraspinal and spinal excitability in response to eccentric training (Duclay et al., 2008), less is known about short-term specific central adaptations after a single bout of ECC, such that the contribution of central factors to RBE is currently debated (Hyldahl et al., 2017).

At the peripheral level, studies have demonstrated changes in fascicle (e.g., reduced elongation) or myotendinous junction behavior (e.g., smaller displacement) during ECC between the first and the second bout partly explaining the reduced amount of EIMD (Lau et al., 2015; Penailillo et al., 2015). Hyldahl et al. (2017) also reported that skeletal muscle extracellular matrix remodeling could increase passive stiffness of the whole muscle the weeks following a bout of ECC to confer protection against a subsequent damaging exercise bout. As a result, muscles could become more resistant to the mechanical stress during the second bout. However, it has also been shown that the RBE can occur without changes in the mechanical properties of muscles (Hoffman et al., 2016). These divergent results may be associated with the fact that muscles are heterogeneously stretched during ECC due to differences in their anatomical (i.e., mono- and bi-articular muscles) properties, leading to inter-muscle variability in muscle damage (Green et al., 2012; Maeo et al., 2018). Ultimately, this may result in different mechanical behavior between muscles within the same muscle group after a second bout of ECC affecting the force generating capacity.

Shear-wave elastography (SWE) is a reliable quantitative real-time method (Gennisson et al., 2010) to assess both passive and active mechanical properties of muscles allowing to calculate an index of stiffness (i.e., shear elastic modulus; µ) of individual muscles in pathological (Bachasson et al., 2018) and physiological conditions (Siracusa et al., 2019). In response to EIMD, resting muscle µ is increased early (<1 h) and may remain elevated during the days following ECC (Lacourpaille et al., 2014; Lacourpaille et al., 2017; Heales et al., 2018; Ema et al., 2021). As Lacourpaille et al. (2017) found that the increase in muscle µ was associated to the magnitude of force loss measured at 48 h, muscle µ has been used an indicator of EIMD. Mechanisms such as calcium homeostasis disturbance due to sarcolemma disruption (Proske and Morgan, 2001), as well as the muscle volume changes due to edema exerting strain on the perimysium and epimysium connective tissues (Whitehead et al., 2001), could be involved in this increased µ after EIMD. A study has also reported an increased resting µ up to 21 days after an ECC of the elbow flexors (Lacourpaille et al., 2014). Whether this reflects the protective mechanical adaptation conferred by the RBE remains unknown (Hyldahl et al., 2017). To date, no study has evaluated the sensibility of the resting µ to RBE.

Therefore, this study was designed to determine whether the magnitude and time-course of the quadriceps resting µ would be differently affected after two identical eccentrically-biased exercises in healthy individuals. We hypothesized that resting µ would be increased to a smaller extent and for a shorter duration after the second damaging exercise bout compared to the same bout performed 2 weeks earlier. The first bout would confer protection against extensive muscle damage through an adaptation of the muscle stiffness before the second bout (i.e., higher resting µ). Moreover, we expected that the magnitude of the adaptive response would differ between the quadriceps muscles (*vastus lateralis vs rectus femoris*) owing to their different anatomical and architectural properties.

## Materials and Methods

### Participants

Sixteen young males (age: 31.5 ± 5.9 years, body mass: 77.1 ± 11.1 kg, height: 1.80 ± 0.06 m, body mass index: 23.9 ± 2.4 kg m^−2^, and fat mass: 15.9 ± 5.4%) were fully informed of the experimental procedures, gave their written informed consent and agreed to participate in this study. Participants performed regular activity (between 3 and 8 h.week^−1^), had no recent history of muscular, joint, or bone disorders and did not take any medication that could affect NM responses. None of them experienced strenuous mountain trekking and/or downhill walking (DW) and none had been involved in a resistance-training program in the past 6 months. Participants were instructed not to perform unaccustomed activities and any interventions that may interfere with recovery such as massage, icing, nutritional supplementation and nonsteroidal anti-inflammatory drugs during the experimental period. Each participant realized an inclusion session, consisting of a complete medical examination, including the collection of anthropometric data and complete familiarization with the experimental NM and SWE procedures. The study was conducted in accordance with the Declaration of Helsinki (Schuklenk, 2001) and was approved by the Regional Ethics Committee (CPP Ile-de-France 8, France, registration number: 2019-A01210-57, SMILE).

### Protocol

**FIGURE 1:**
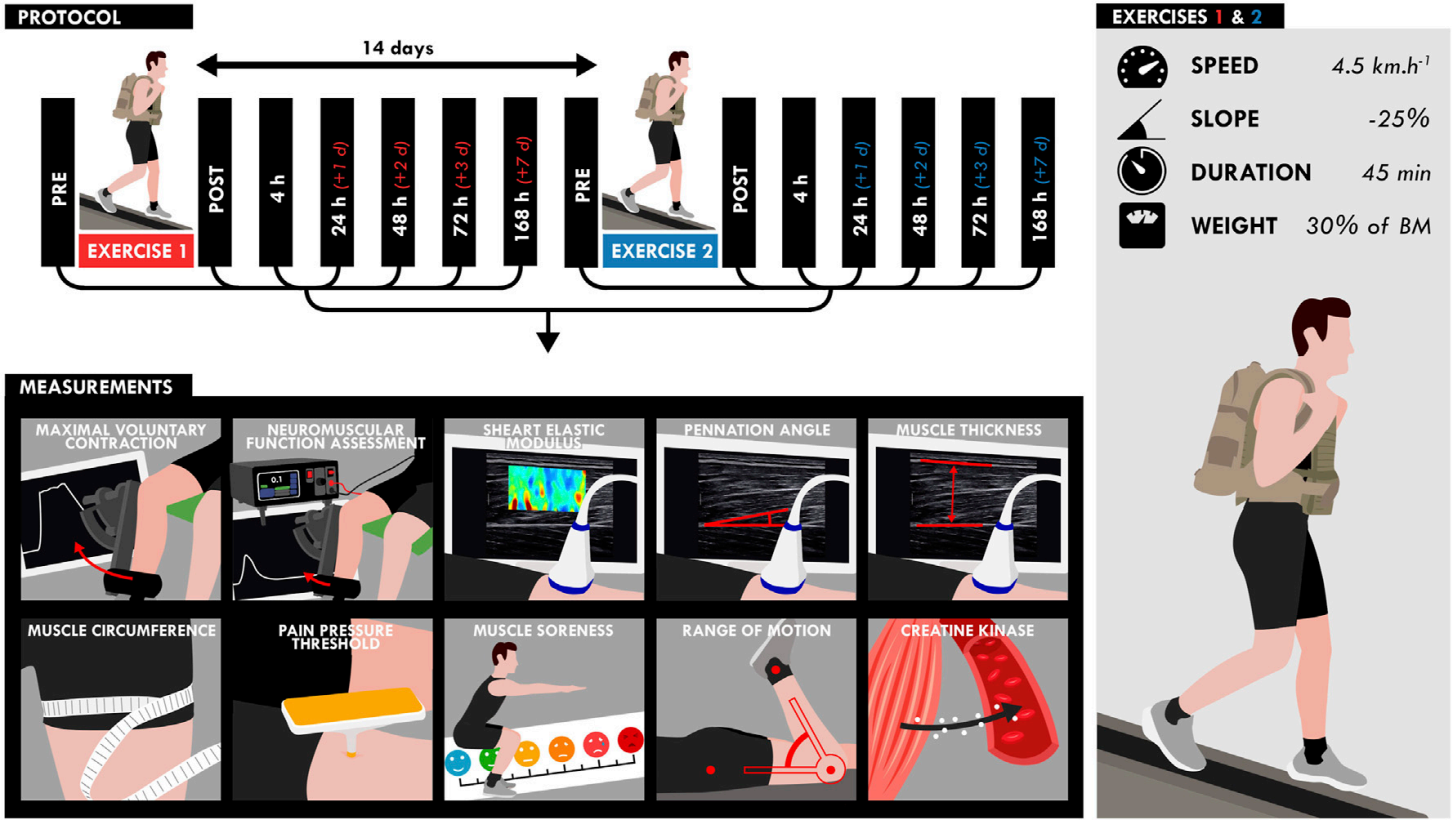
Design of the study. Participants performed two bouts separated by 2 weeks of downhill walking on a treadmill for 45 min while carrying a load equivalent to 30% of the body mass (treadmill gradient: 25%; speed: 4.5 km.h^−1^). All measurements (bottom) were assessed at different time points as shown on the timeline (top).

Participants performed two 45-min DW bouts separated by 2 weeks on a treadmill (pulsar 3p 4.0, HP cosmos, Warwickshire, United Kingdom) while carrying a load equivalent to 30% of the body mass (carrying: a weighted vest: 10% of the body mass; a backpack: 20% of the body mass) (Figure 1). The treadmill gradient was set to −25%, velocity to 4.5 km.h^−1^ and the participants were instructed to walk at their own preferred stride length and frequency. Five participants were not able to finish the walking bout and performed the first exercise to exhaustion (30.2 ± 8.6 min). These participants performed the same exercise duration during the second exercise. NM assessments (voluntary and evoked torque), quadriceps muscle soreness, knee range of motion (ROM), muscle architecture and resting shear elastic modulus (µ) of the *vastus lateralis* (VL) and *rectus femoris* (RF) muscles measured by SWE were assessed on the right limb before (PRE), within 1 h (POST), and the hours/days (4, 24, 48, 72, and 168 h) after the two DW bouts. Blood samples were obtained before, immediately after (POST), and 2, 6, 24, 48, 72 and 168 h (7 days) after the two exercise bouts to analyze serum creatine kinase (CK) activity.

## Data Collection

### Traditional EIMD Symptoms

#### Neuromuscular Function Assessments

Voluntary and evoked torques were measured using an isokinetic dynamometer (Cybex Norm, Lumex, Ronkonkoma, NY, United States). Participants were comfortably positioned on an adjustable chair with the hip joint flexed at 70° (0° = neutral supine position). The axis of rotation of the dynamometer was aligned with the femoral condyles of the femur and the lever arm was attached 1–2 cm above the malleolus with a Velcro strap. All measurements were taken from the participant’s right leg (knee angle = 90°; 0° = knee fully extended). Torque data were corrected for gravity, digitized and exported at a rate of 2 kHz to an external analog-to-digital converter (Powerlab 16/35; ADInstruments, New South Wales, Australia) driven by the Labchart pro 8.1 software (ADInstruments, New South Wales, Australia). During each MVC, the participants were instructed to grip the lateral handles of the seat in order to stabilize the pelvis and were strongly encouraged by the investigator to push as fast and hard as possible.

Potentiated single (Twitch) and double electrical stimulations delivered at 10 and 100 Hz (Db10 and Db100, respectively) at rest. Briefly, quare-wave pulses with a width of 1 m at a maximal voltage of 400 V were delivered percutaneously to the femoral nerve using an electrical stimulator (Digitimer DS8R, Welwyn Garden City, United Kingdom) connected to a cathode (5 × 5 cm; Axelgaard manufacturing, Fallbrook, United States) placed over the femoral nerve in the femoral triangle and an anode (5 × 9 cm; Axelgaard manufacturing, Fallbrook, United States) placed on the gluteal fold. Intensity of stimulation ranged from 65 to 260 mA and corresponded to 130% of the optimal intensity (i.e., the intensity at which maximal non-potentiated single twitches started to plateau). Intensities were determined from single pulses delivered from 40 to 200 mA in 5-mA increments.

#### Blood Collection

Approximately 3.5 ml of blood was drawn from the antecubital vein into a serum separating tube (SST; 5 ml, Becton Dickinson vacutainer, Franklin Lakes, United States).

#### Muscle Soreness

The magnitude of muscle soreness of the whole quadriceps was assessed using a visual analog scale, consisting of a 100-mm line representing “no pain” at one end (0 mm), and “very, very painful” at the other (100 mm), while performing a squat over a 90°-knee range of motion.

#### Muscle Pain Pressure Threshold

An algometer (NOD, OT Bioelettronica, Turino, Italy) with a rigid plastic rod covered by a rubber surface with an area of 1.0 cm^2^ and a scale ranging from 0 to 500 kPa was used to measure muscle pain pressure threshold (PPT). First, the procedures were explained clearly to the subject. The participants were evaluated in a sitting position with the knee placed at 90° of flexion (0° = knee fully extended), in a relaxed state. The compression pressure was applied on the mid portion of the VL and RF muscles. The pressure was applied vertically and it increased at a constant rate of 50 kPa per seconds. Participants were asked to say “stop” when they began to feel muscle pain or discomfort. Two repetitive measurements were performed for each muscle at an interval of 60 s.

#### Range of Motion

The passive knee ROM was measured using a universal goniometer marked in 1° increments. ROM assessment consisted of placing the participant prone with the hip at 0° of flexion. The femur was stabilized to avoid any hip flexion movement. The goniometer was aligned with the right lateral femoral epicondyle. The proximal part of the goniometer was placed along the lateral aspect of the femur using the greater trochanter as a reference and the distal part was placed along the fibula using the lateral malleolus as a reference. The starting position was fixed at 0° (knee fully extended) and ROM was measured while the investigator moved slowly the knee into flexion until the participant reported muscle soreness. To take into account of the effect of muscle thixotropy (Lakie and Campbell, 2019), measurements were performed six times for the right leg and the average value of the fifth and the sixth measurements was used for data analysis. ROM was considered as an indicator of joint stiffness.

#### Thigh Circumference

Circumference was measured at the mid portion of the right thigh (midpoint between the greater trochanter of the femur and the lateral femoral epicondyle) while the participant in a standing position with the feet placed shoulder-width apart. Thigh was marked at the mid portion with a horizontal line using a permanent marker and measured to the nearest 0.1 cm using a tape. Thigh circumference was considered as an indicator of thigh muscles swelling.

### Ultrasound Measurements

An ultrafast ultrasound scanner (Aixplorer version 12.2; Supersonic Imagine, Aix-en-Provence, France) coupled with a linear transducer array (SuperLinear 15–4; Supersonic Imagine, Aix-en-Provence, France) was used in both SWE (musculoskeletal preset, penetration, no persistence) and research modes, as previously proposed (Bercoff et al., 2004). The B-mode ultrasound was first set to determine the optimal transducer location and maximize the alignment between the transducer and the direction of the RF and VL muscle fascicles. Transducer alignment was considered correct when muscle fascicles and aponeurosis could be delineated across the image without interruption. The transducer was then fixed at 50% of the total muscle length respectively using a dynamic probe fixation device (with 360° adjustments, USONO, Eindhoven, Netherlands) placed over the skin, which was coated with a water-soluble transmission gel (Aquasonic, Parker laboratory, Fairfield, NJ, United States) to ensure acoustic coupling. This fixation allows to avoid any movement which could affect the position and the orientation of the probe, and avoid excessive pressure applied to the muscle. The position of the probe was marked on the skin using a permanent marker to ensure the same positioning along the experiment.

A fixed-size rectangular region of interest (ROI), i.e., the region in which shear-wave propagation was analyzed within the muscle, was placed in the middle of the B-mode image below the superficial aponeurosis within the VL and RF muscle belly. The position of the ROI was carefully located on the B-mode image during the familiarization session to be sure to keep the same ROI along the experiment. 5-s SWE sequences (frame rate: 1–2 Hz) were performed at rest for VL and RF muscles with the knee joint positioned at 90° and 120° (0° = knee fully extended).

## Data Analysis

### Traditional EIMD Symptoms

#### Neuromuscular Function Assessments

Delayed (i.e., >24 h) NM function impairment and specifically maximal voluntary contraction (MVC) torque loss is usually considered as the most reliable and valid marker for the evaluation of the magnitude of EIMD (Warren et al., 1999; Paulsen et al., 2012). MVC torque was determined as the peak torque reached during the maximal effort.

Contractile properties of the knee extensors muscles were assessed by determining the amplitude of the responses to potentiated single (Twitch) and double electrical stimulations delivered at 10 and 100 Hz (Db10 and Db100, respectively) at rest (Verges et al., 2009). The Db10 Hz-to-Db100 Hz ratio was calculated and used to assess changes in excitation-contraction (E-C) coupling. The double pulse (at 100 Hz) superimposition technique, based on the interpolated-twitch method (Merton, 1954), enabled us to estimate the maximal voluntary activation level (VAL). The VAL (Merton, 1954) was computed as follows:

$$VAL=\left(\frac{1-superimposed\;Db100}{potentiated\;Db100}\right)\times 100$$

Twitch rate of torque development (RTD) was calculated as the slope of the torque-time curve between the onset of torque development and the peak twitch torque occurring after a single electrical stimulation, and reflect the ability of muscle to rapidly produce torque (Maffiuletti et al., 2016). It should be noted that RTD could be influenced by muscle stiffness (Maffiuletti et al., 2016).

#### Creatine Kinase Activity

SST tube was allowed to clot at ambient temperature for 30 min, and centrifuged (2,000 g, 4°C, 10 min). One mL of serum was collected and frozen at −80°C until further analyses. CK activity was analyzed from 200 µl of serum using an auto analyzer (Advia 1,800; Siemens Healthineers, Erlangen, Germany).

#### Muscle Pain Pressure Threshold

The PPT test determines the minimal amount of pressure over a given area in which a steadily increasing painless stimulus turns into a painful sensation. The mean value of the two measurements was used for analysis.

#### Muscle Thickness

Muscle thickness (MT) of the RF and VL muscles was assessed from three B-mode images obtained by using the Aixplorer device at knee angle of 90° (0° = knee fully extended). B-mode images were exported to a workstation and analyzed *off-line* using the ImageJ software (version 1.42, National Institute of health, Bethesda, MA, United States). Briefly, calibration was performed on the sofware using the time Gain Compensation (TGC) that automatically adjust the B-mode gain at different depths and for different tissue attenuations provided by the ultrasound scanner (Aixplorer version 12.2; Supersonic Imagine, Aix-en-Provence, France). Overall brightness is also automatically adjusted depending on setting of auto TGC. MT was calculated as the length (in cm) between the superficial and depth aponeurosis. MT was calculated at distal, middle, and proximal part of the B-mode image and the average value of these three lengths was used for further analysis. MT was considered as an indicator of muscle swelling.

### Shear-Wave Elastography Measurements

**FIGURE 2:**
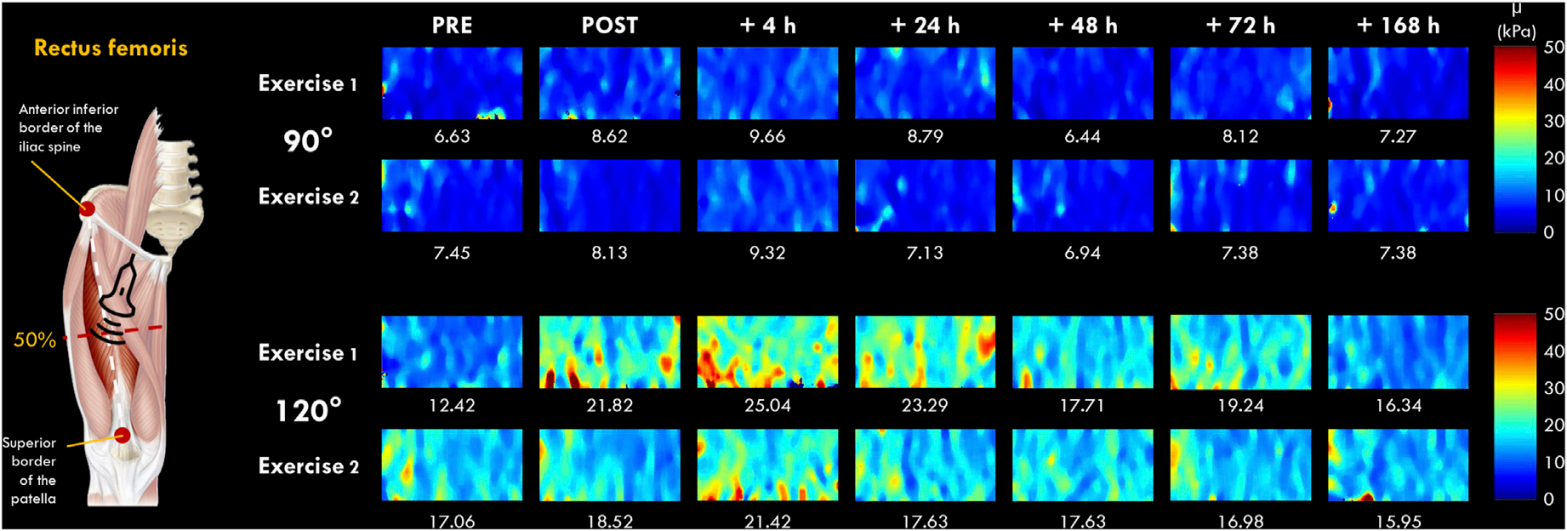
Typical two-dimensional real-time muscle shear modulus color maps of the *rectus femoris* muscle obtained with a frame rate of 1–2 Hz and a spatial resolution of 1 × 1 mm for 90 and 120° of knee flexion (0° = full extension). Values under the color maps were the mean value of the region of interest placed in the middle of the B-mode image below the superficial aponeurosis within the RF muscle belly.

A two-dimensional real-time shear wave velocity (in m.s^−1^) color map with a spatial resolution of 1 × 1 mm was obtained, as previously described (Siracusa et al., 2019; Chalchat et al., 2020) (Figure 2). The shear wave velocity raw data was then transferred to a workstation, converted to shear elastic modulus (µ) and analyzed using a MATLAB script developed in our laboratory (MathWorks, Natick, MA, United States). The µ was obtained as follows:

$$\text{µ}=\rho .Vs^{2}$$

where *ρ* is the muscle density (1,000 kg.m^−3^) and *V*_*s*_ is the shear-wave velocity (in m.s^−1^).

The µ values were averaged over the ROI (a ∼36 × 114 matrix) without the empty (0 m.s^−1^) and saturated (16.32 m.s^−1^) values, and the average of 5-8 consecutive reconstructed images from raw data (available for Aixplorer version 12.2 with research pack) was used for subsequent analyses. As previously described by Lacourpaille et al. (2017), the slope of the relationship between the change in µ and the knee joint angle of 90° and 120° at each time point (PRE, POST, 4, 24, 48, 72 h and 7 days) was calculated and interpreted as an index of increase in µ (expressed in %). This index allowed us to compare the increase in µ between VL and RF muscles regardless of their relative length (Lacourpaille et al., 2017). We chose to investigate VL and RF muscles due to the difference anatomical properties (e.g., mono-versus bi-articular muscles) (Jacobs and van Ingen Schenau, 1992) and due to their differences in susceptibility to EIMD (Lacourpaille et al., 2017; Heales et al., 2018; Maeo et al., 2018). Raw data are displayed in Supplementary Material S1.

### Pennation Angle

Pennation angle (PA) of the RF and VL muscles were measured from three B-mode images obtained with the ultrasound device. B-mode images were exported to a workstation and analyzed off-line using the ImageJ software (version 1.42, National Institute of health, Bethesda, MA, United States). PA was calculated as the angle (in °) between the deep aponeurosis and the muscle fascicle orientation. Measurements of PA were performed to facilitate the interpretation of µ, which could be affected by muscle architecture (Gennisson et al., 2010; Lacourpaille et al., 2012; Eby et al., 2013; Ewertsen et al., 2016). Indeed, Eby et al. (2013) found that fascicle orientation of 45° relative to ultrasound beam affect substantially shear wave modulus.

### Index of Protection

In order to assess the magnitude of the protective effect conferred by the first bout of DW, an index of protection (IP) was calculated for EIMD symptoms (e.g., NM parameters, muscle µ, CK activity, muscle soreness, PPT, thigh circumference) using an equation adapted from Chen et al. (2012): [relative changes from PRE after the exercise 2 — relative changes from PRE after the exercise 1] with PRE corresponding to 100%. For instance, when the RF µ represent 150% of the PRE value after the first exercise and only 120% after the second exercise, the calculation is: 120—150 = −30. −30% indicates that the increase in RF µ is attenuated by 30% after the second exercise compared to the first. The equation was adapted because the initial version from Chen et al. (2012) was applicable to mean values but not to individual values. Two calculations were performed for IP. The aim was i) to detect the greatest variation from PRE among the values recorded from POST to 7 days (IP for magnitude of changes); ii) to detect the rate of recovery using the final value at 7 days (IP for rate of recovery).

### Statistical Analyses

The data were screened for normality of the distribution and homogeneity of variances using the Shapiro–Wilk normality and Levene tests, respectively. Then, differences in absolute or log-transformed values were analyzed using a one-way repeated measures ANOVA (effect: time) for all parameters. A two-way repeated measures ANOVA (effect: time × exercise) was performed on relative changes from PRE for all parameters, except imagery parameters (three-way: time × exercise × muscle). When no interactions (time × angle × muscle) were found for these parameters, two-way ANOVAs (effect: time × angle) with repeated measures were used to compare relative values from PRE for each muscle (i.e., RF and VL). When the ANOVA revealed significant effects or interactions between factors, Holm post-hoc tests were applied to test for differences between means. As it is not possible to compare PRE values between conditions using relative changes from PRE, we used a *t*-test to identify differences in absolute PRE values between conditions.

When the normality and homogeneity assumptions were violated despite the log transformation, a non-parametric Friedman analysis of variance was performed rather than one-way repeated measures ANOVA (VAL, muscle soreness, ROM and PPT). As two- or three-way repeated measures ANOVA are inappropriate when the assumptions are violated, we used a *t*-test to test the difference in relative changes from PRE between conditions (i.e., exercise and muscle). In this case, Holm corrections for multiple comparisons were used. Finally, Wilcoxon rank test was performed rather than *t*-test when the normality was violated.

Correlations were performed between relative changes (i.e., percentage of the initial value) in muscle µ measured 14 days after the first exercise (i.e., from PRE exercise 1 to PRE exercise 2) and both IP of EIMD symptoms (NM parameters and muscle µ, CK activity, soreness, swelling), to investigate whether long term (i.e., 14 days) changes in muscle µ are implicated in the RBE. Indeed, as the RBE is characterized by smaller and shorter alteration of EIMD symptoms after the second bout (Chen et al., 2012; Hyldahl et al., 2017), we chose to test correlations using IP of peak changes (i.e., magnitude of EIMD reduction) and IP of changes at 7 days (i.e., changes in rate of recovery of EIMD symptoms). The level of association was assessed using Pearson correlation coefficient (r) or using Spearman’s rank correlation coefficient (*ρ*) when the normality and homogeneity assumptions were violated.

Results with a *p*-value < 0.05 were considered significant. Statistical procedures were performed using Jamovi (The jamovi project (2020). jamovi (Version 1.2) [Computer Software]. Retrieved from https://www.jamovi.org, Sydney, Australia). Missing data were imputed using the missMDA R package. The results presented are expressed as means ± SD. Additional information about statistical analyses were displayed in Supplementary Material S2.

## Results

### PRE Exercise Measurements

The statistical analyses showed no significant differences between exercise 1 and exercise 2 for absolute PRE values of all parameters, except for RF µ and VL PA, which were higher before exercise 2 (Table 1).

**TABLE 1:** Absolute PRE values of Exercise 1 and Exercise 2 for all parameters. MVC: maximal voluntary torque; VAL: voluntary activation level; RTD: rate of torque development; Db10: doublet at 10 Hz; Db100: doublet at 100 Hz; RF: *rectus femoris*; VL: *vastus lateralis*; PPT: pain pressure threshold. Results with a *p*-value < 0.05 are considered significant and are displayed in bold characters.

Absolute PRE values	Exercise 1			Exercise 2			*p*
MVC force (N.m)	314.3	±	49.7	311.2	±	70.3	0.81
VAL (%)	96.6	±	2.4	96.4	±	2.2	0.79
RTD (N.m.s^−1^)	1,137	±	261	1,067	±	277	0.27
Twitch (N.m)	67.5	±	14.7	65.8	±	14.5	0.35
Db10 (N.m)	105.4	±	22.9	103,0	±	23.6	0.45
Db100 (N.m)	100.1	±	19.6	99.3	±	18.6	0.77
Db10-to-Db100 Hz ratio	1.05	±	0.13	1.06	±	0.14	0.95
Creatine kinase (U/L)	151	±	11	151	±	13	0.43
Muscle Soreness (cm)	0.2	±	0.6	0.0	±	0.9	0.69
RF PPT (kPa)	415	±	127	406	±	129	0.30
VL PPT (kPa)	451	±	88	433	±	91	0.07
Range of motion (°)	150.7	±	10.8	151.5	±	12.7	0.61
Thigh Circumference (cm)	57.5	±	3.7	57.3	±	3.3	0.42
RF muscle thickness (cm)	2.46	±	0.35	2.58	±	0.38	0.29
VL muscle thickness (cm)	2.34	±	0.41	2.43	±	0.42	0.09
RF pennation angle (°)	12.9	±	2.7	13.8	±	3.8	0.28
VL pennation angle (°)	9.7	±	2.7	12.2	±	4.1	**0.02**
RF shear modulus (slope)	0.44	±	0.26	0.57	±	0.27	**<0.001**
VL shear modulus (slope)	0.18	±	0.09	0.2	±	0.09	0.33

### Traditional EIMD Symptoms

#### Neuromuscular Function Assessments

**FIGURE 3:**
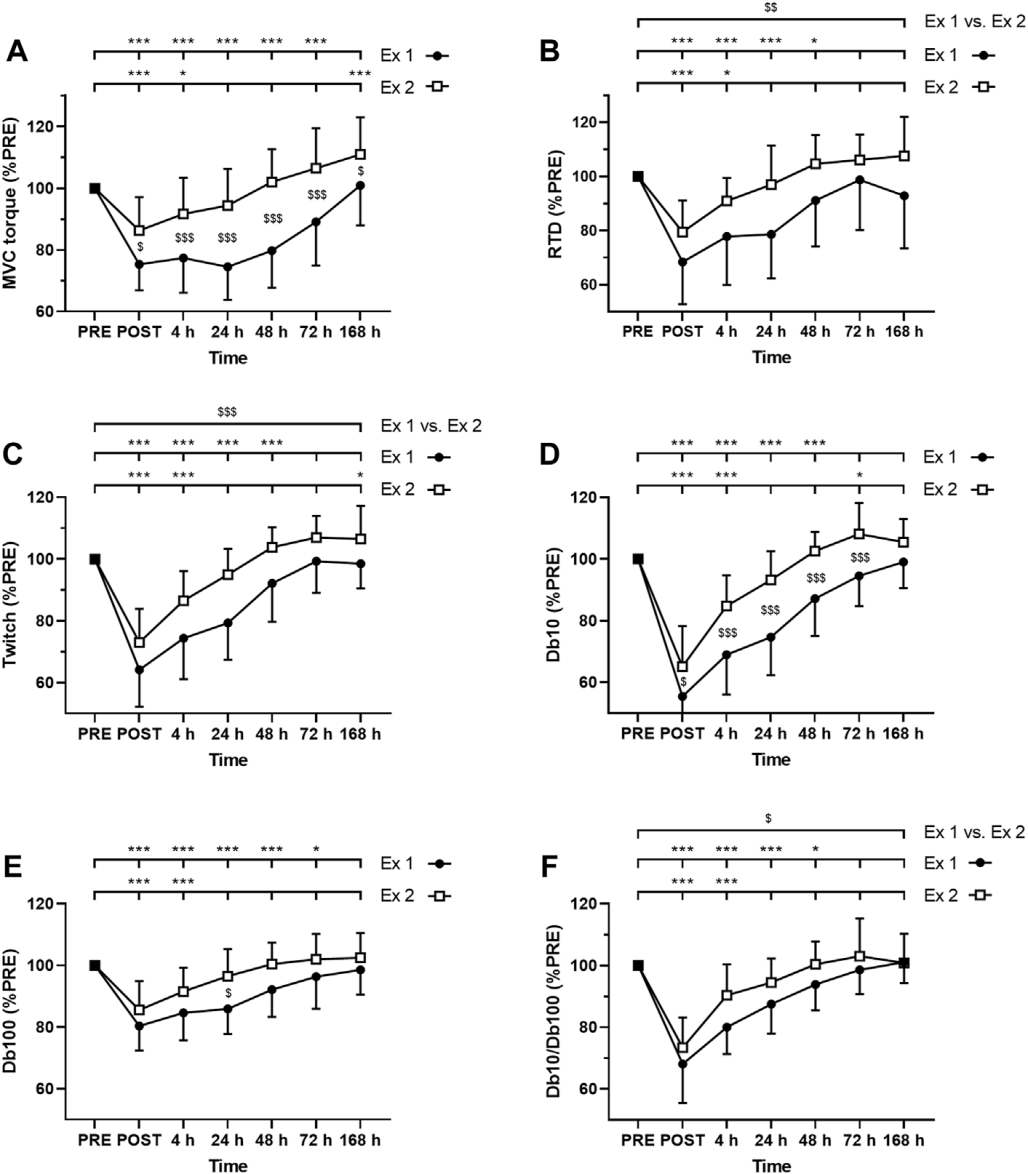
Time course of **(A)** maximal voluntary contraction (MVC) torque **(B)** rate of torque development **(C)** twitch amplitude **(D)** amplitude of the doublet at 10 Hz (Db10) **(E)** amplitude of the doublet at 100 Hz (Db100) and **(F)** Db10 Hz-to-Db100 Hz ratio (Db10/Db100). *, ** and *** correspond to significant difference from PRE value at *p* < 0.05, *p* < 0.01 and *p* < 0.001, respectively. $, $$ and $$$ correspond to significant difference between exercise 1 and exercise 2 at *p* < 0.05, *p* < 0.01 and *p* < 0.001, respectively.

As expected, ANOVA revealed a time effect for absolute values of MVC torque, twitch, Db10, Db100, and Db10-to-Db100 Hz ratio after exercise 1 and exercise 2 (*p* < 0.001; Figure 3). Moreover, significant interactions (time × exercise) were found for MVC torque, Db10 and Db100. The Friedman test also revealed a time effect for absolute values of VAL after the exercise 1 (*p* < 0.001) but not after the exercise 2 (*p* = 0.79).

MVC torque was significantly decreased up to 72 h after the exercise 1 whereas it was decreased up to 4 h after the exercise 2 (Figure 3A). Moreover, post-hoc analysis revealed a smaller decrease in MVC torque after the exercise 2 compared to the exercise 1 at all measurement times (Figure 3A).

VAL was significantly decreased up to 72 h after the exercise 1 whereas no significant changes were found after the exercise 2. Moreover, greater changes of VAL were found after the exercise 1 compared to the exercise 2 at POST (−6.8 ± 4.8% vs −1.9 ± 3.7%; *p* = 0.010) and 48 h (−5.1 ± 4.1% vs. +0.2 ± 2.5%; *p* < 0.001) post-exercise, but not at 4 h (−4.3 ± 5.4% vs. −0.7 ± 3.5%; *p* = 0.072), 24 h (−4.5 ± 4.4% vs.−1.5 ± 2.5%; *p* = 0.099), 72 h (−1.3 ± 2.7% vs. +0.3 ± 2.2%; *p* = 0.152) and 7 days (+0.2 ± 2.5% vs. +0.3 ± 1.9%; *p* = 0.913).

RTD was significantly decreased up to 48 h after the exercise 1 whereas it was decreased up to 4 h after the exercise 2 (Figure 3B). Moreover, the average decrease of RTD during the days following exercise was smaller after the exercise 2 compared to the exercise 1 (Figure 3B).

Similarly, twitch amplitude was significantly decreased up to 48 h after the exercise 1 whereas it was decreased up to 4 h after the exercise 2 (Figure 3C). Moreover, the average decrease of twitch amplitude during the days following exercise was smaller after the exercise 2 compared to the exercise 1 (Figure 3C).

Db10 amplitude was significantly decreased up to 48 h after the exercise 1 whereas it was decreased up to 4 h after the exercise 2 (Figure 3D). Moreover, Db10 amplitude was significantly less decreased after the exercise 2 compared to the exercise 1 at POST to 72 h post-exercise (Figure 3D).

Db100 amplitude was significantly decreased up to 72 h after the exercise 1 whereas it was decreased up to 4 h after the exercise 2 (Figure 3E). Moreover, Db100 amplitude was significantly less decreased 24 h after the exercise 2 compared to the exercise 1 (Figure 3E).

Db10-to-Db100 Hz ratio was significantly decreased up to 48 h after the exercise 1 whereas it was decreased up to 4 h after the exercise 2 (Figure 3F). Moreover, the average decrease of the Db10-to-Db100 Hz ratio during the days following exercise was smaller after the exercise 2 compared to the exercise 1 (Figure 3F).

#### Creatine Kinase

**FIGURE 4:**
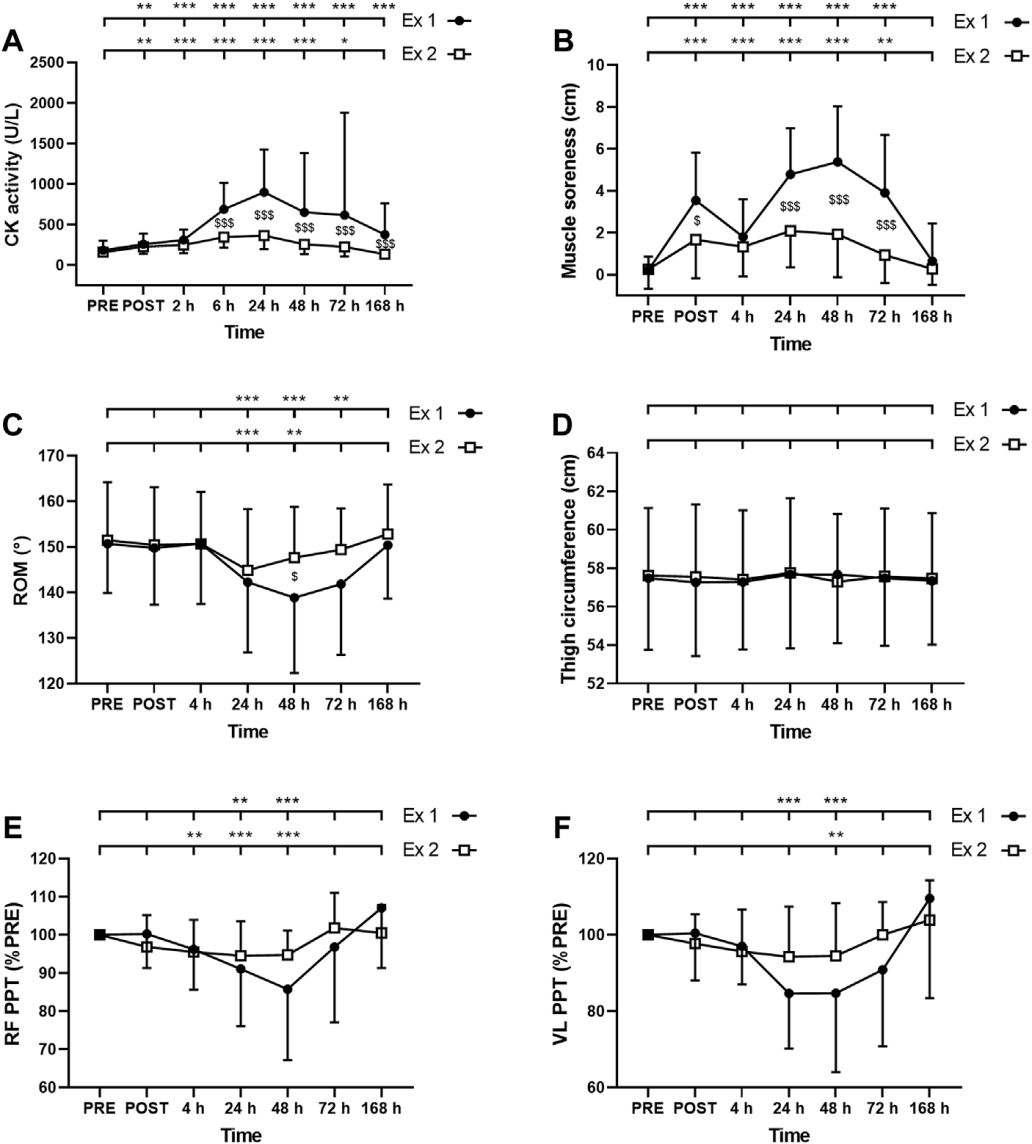
Time course of **(A)** serum creatine kinase (CK) activity **(B)** muscle soreness **(C)** range of motion (ROM), thigh circumference **(D)**, and pain pressure threshold (PPT) for **(E)** *rectus femoris* (RF) and **(F)** *vastus lateralis* (VL) for exercise 1 and 2. *, ** and *** correspond to significant difference from PRE value at *p* < 0.05, *p* < 0.01 and *p* < 0.001, respectively. $, $$ and $$$ correspond to significant difference between exercise 1 and exercise 2 at *p* < 0.05, *p* < 0.01 and *p* < 0.001, respectively.

ANOVA revealed a time effect for absolute values of CK activity after exercise 1 and exercise 2 (*p* < 0.001). CK activity was increased up to 7 days and up to 72 h after the exercise 1 and 2, respectively (Figure 4A). Moreover, a significant interaction effect (time × exercise) was found for this parameter (*p* < 0.001). Post-hoc analysis revealed a smaller increase in this parameter after the second exercise compared to the first exercise at 6–168 h (7 days) post-exercise (Figure 4A).

#### Muscle Soreness

The Friedman test showed a significant effect of time for muscle soreness after the exercise 1 and the exercise 2 (*p* < 0.001). Muscle soreness was increased up to 72 h after both exercise 1 and exercise 2 (Figure 4B). Moreover, a greater increase in muscle soreness was found after the exercise 1 compared to the exercise 2 at POST, 24, 48, and 72 h post-exercise (Figure 4B).

#### Range of Motion

The Friedman test showed a significant effect of time for ROM after the exercise 1 and the exercise 2 (*p* < 0.001). ROM was decreased between 24 and 72 h after the exercise 1 and between 24 and 48 h after the exercise 2 (Figure 4C). ROM was decreased by a smaller amplitude after the second exercise compared to the first exercise at 48 h post-exercise (Figure 4C).

#### Thigh Circumference

ANOVA showed no significant effect of time (*p* = 0.10); exercise (*p* = 0.47) or interaction (time × exercise; *p* = 0.08) for thigh circumference (Figure 4D).

#### Pain Pressure Threshold

The Friedman test showed a significant effect of time for both RF and VL PPT after the exercise 1 and the exercise 2 (*p* < 0.001). RF PPT was decreased at 24 and 48 h after the exercise 1 and between 4 and 48 h after the exercise 2 (Figure 4E). VL PPT was decreased at 24 and 48 h after the exercise 1 and only at 48 h after the exercise 2 (Figure 4F). Three-way ANOVA did not show any muscle effect (*p* = 0.451).

#### Muscle Thickness

**FIGURE 5:**
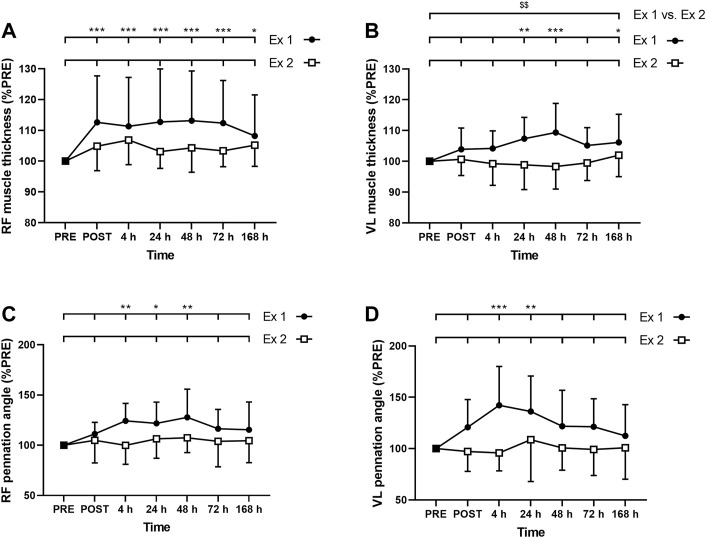
Time course of **(A)** *rectus femoris* (RF) and **(B)** *vastus lateralis* (VL) muscle thickness, and **(C)** RF and **(D)** VL pennation angle for exercise 1 and exercise 2. *, ** and *** correspond to significant difference from PRE value at *p* < 0.05, *p* < 0.01 and *p* < 0.001, respectively. $$: significant difference between exercise 1 and exercise 2 at *p* < 0.01.

ANOVA revealed a time effect for absolute RF MT after exercise 1 (*p* < 0.001) but not after exercise 2 (*p* = 0.062). Similarly, ANOVA revealed a time effect for absolute VL MT after exercise 1 (*p* = 0.02) but not after exercise 2 (*p* = 0.431). RF MT was increased up to 7 days after the exercise 1 (Figure 5A), whereas VL was increased at 24, 48 h and 7 days (Figure 5B). Moreover, the average increase of the MT during the days following exercise was smaller after the exercise 2 compared to the exercise 1 for VL (*p* = 0.009; Figure 5B) but not for RF (RF: *p* = 0.094; Figure 5A). The three-way ANOVA showed that RF MT was, on average, more increased than VL MT (*p* < 0.001).

### Pennation Angle

ANOVA revealed a time effect for absolute RF PA after exercise 1 (*p* = 0.006) but not after exercise 2 (*p* = 0.697). Similarly, ANOVA revealed a time effect for absolute VL PA after exercise 1 (*p* = 0.003) but not after exercise 2 (*p* = 0.758). RF PA was increased at 4, 24, and 48 h after the exercise 1 (Figure 5C), whereas VL PA was increased at 4 and 24 h (Figure 5D). Three-way ANOVA did not show any muscle effect (*p* = 0.491).

### Muscle Shear Modulus (µ)

**FIGURE 6:**
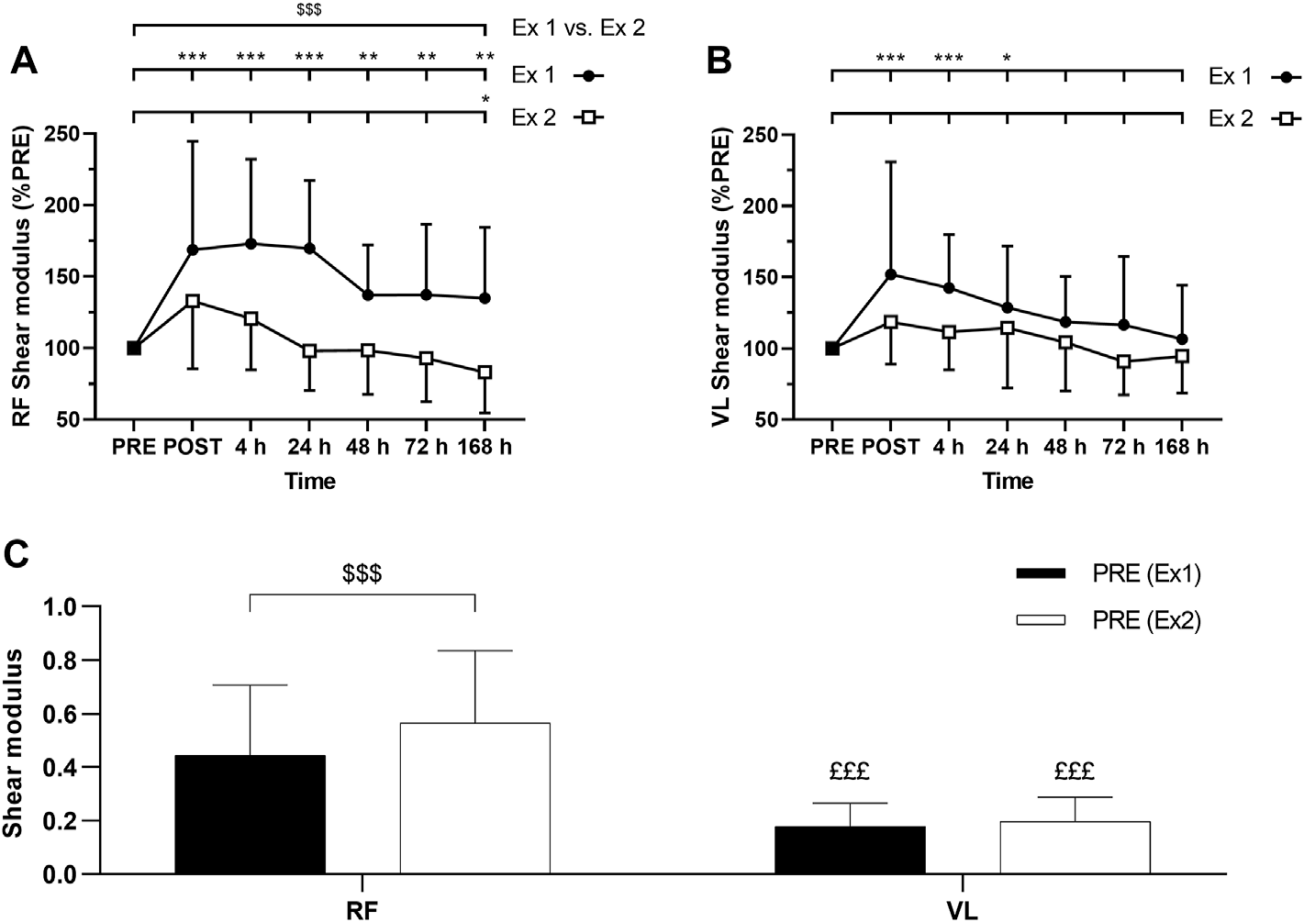
Time course of **(A)** *rectus femoris* (RF) and **(B)** *vastus lateralis* (VL) shear modulus expressed in percentage of PRE value for exercise 1 and 2, and **(C)** absolute values of RF and VL shear modulus (slope) at PRE for exercise 1 and exercise 2. Ex: Exercise. *, ** and *** correspond to significant difference from PRE value at *p* < 0.05, *p* < 0.01 and *p* < 0.001, respectively. $$$: significant difference between exercise 1 and exercise 2 at *p* < 0.001, £££: significantly different from RF value at *p* < 0.001.

ANOVA revealed a time effect for absolute RF µ after exercise 1 and exercise 2 (*p* < 0.001). Similarly, ANOVA revealed a time effect for absolute VL µ after exercise 1 (*p* < 0.001) and exercise 2 (*p* = 0.01). RF µ was increased up to 7 days after the exercise 1 (Figure 6A). VL µ was transiently increased up to 24 h after exercise 1 whereas no significant changes were found after the exercise 2 (Figure 6B). The average increase of relative changes in RF µ from PRE during the days following exercise was smaller after the exercise 2 compared to the exercise 1 (Figure 6A). However, relative changes in VL µ from PRE were not significantly different between the exercises (Figure 6B). No significant interactions (time × exercise) were found for RF and VL µ.

Absolute RF µ PRE value was significantly greater for the exercise 1 compared to exercise 2 (Figure 6C). Such a difference was not observed for absolute VL µ PRE values between exercise 1 and exercise 2 (Figure 6C). Absolute µ PRE values were significantly higher for RF compared to VL for exercises 1 and 2 (Figure 6C). Moreover, the average increase of relative changes in µ from PRE during the days following exercise was smaller for VL compared to RF after the exercise 1 (*p* = 0.04) but not after the exercise 2 (*p* = 0.87).

### Correlation Between Resting µ at 14 days and Index of Protection

**FIGURE 7:**
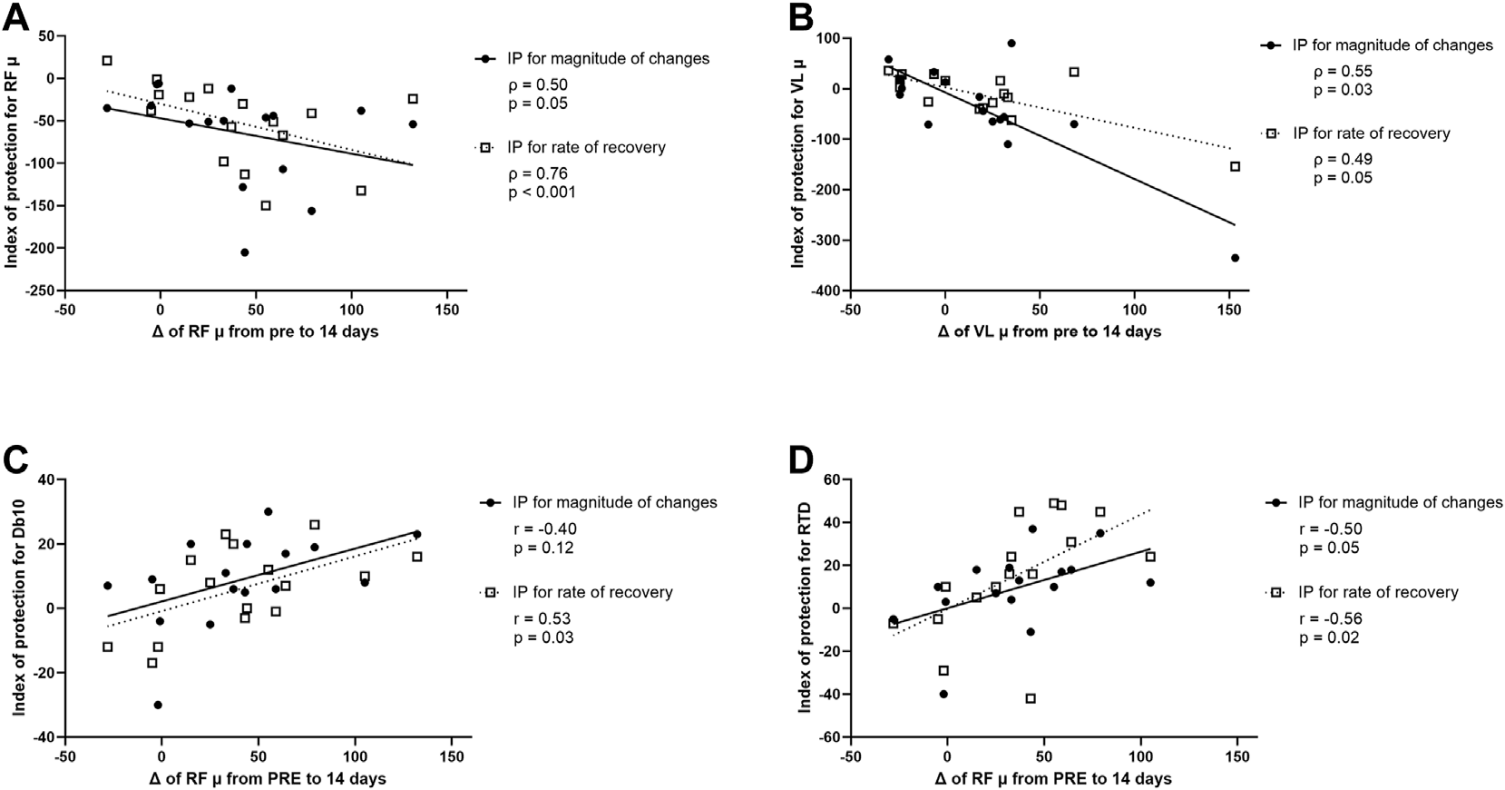
**(A)** Correlation between modifications (Δ) of *rectus femoris* (RF) muscle shear elastic modulus (µ) from PRE to 14 days after the first exercise (i.e., from PRE exercise 1 to PRE exercise 2) and index of protection (IP) for magnitude of changes (filled circle) or rate of recovery (open square) for RF µ. **(B)** Correlation between Δ of *vastus lateralis* (VL) µ from PRE to 14 days after the first exercise and IP for magnitude of changes (filled circle) or rate of recovery (open square) for VL µ. **(C)** Correlation between Δ of RF µ from PRE to 14 days after the first exercise and IP for magnitude of changes (filled circle) or rate of recovery (open square) for the amplitude of the doublet at 10 Hz (Db10). **(D)** Correlation between Δ of RF µ from PRE to 14 days after the first exercise and IP for magnitude of changes (filled circle) and rate of recovery (open square) for the rate of torque development (RTD).

A correlation was found between relative changes in RF µ measured 14 days after the first exercise (i.e., from PRE exercise 1 to PRE exercise 2), and the IP for RF µ for peak changes and changes at 7 days post-exercise (Figure 7A). The greater the increase of RF µ 14 days after the first exercise, the smaller the increase of RF µ after the second exercise bout compared to the first bout (for both peak changes and changes at 7 days; Figure 7A). Similarly, a correlation was found between relative changes in VL µ measured 14 days after the first exercise, and the IP for VL µ for peak changes and changes at 7 days post-exercise (Figure 7B). The greater the increase in VL µ 14 days after the first exercise, the smaller the increase of VL µ after the second exercise bout compared to the first bout (for both peak changes and changes at 7 days; Figure 7B).

A correlation was also found between relative changes in RF µ measured 14 days after the first exercise and the IP for Db10 for changes at 7 days post-exercise but not for peak changes (Figure 7C). The greater the increase of RF µ 14 days after the first exercise, the smaller the decrease of Db10 after the second exercise bout compared to the first bout (only for changes at 7 days; Figure 7C). Finally, a significant correlation was found between relative changes in RF µ measured 14 days after the first exercise and the IP for RTD and the IP for RF µ for peak changes and changes at 7 days post-exercise (Figure 7D). The greater the increase of RF µ 14 days after the first exercise, the smaller the decrease of RTD after the second exercise bout compared to the first bout (for both peak changes and changes at 7 days; Figure 7D).

No significant correlation was found between relative changes in RF and VL µ measured 14 days after the first exercise and both IP for other NM parameters and EIMD symptoms (CK activity, muscle soreness, PPT, thigh circumference).

## Discussion

This study was designed to determine whether the resting µ of the quadriceps muscle group (VL and RF muscles) is sensitive to the RBE. We observed that the resting µ increased to a smaller extent and for a shorter duration after a second bout of eccentric-biased exercise compared to the same bout performed 2 weeks earlier confirming therefore the hypothesis that the first bout of DW exercise confers an adaptation of the RF muscle stiffness. Indeed, RF µ remained elevated 2 weeks after the end of the first exercise (i.e., before the second bout), when EIMD symptoms disappeared. However, the adaptation was not observed for VL µ, confirming the hypothesis that the adaptive response differs between the RF and VL. Moreover, we found that the increase in RF muscle stiffness observed 14 days after the first exercise bout was correlated with the functional protection conferred by the RBE (i.e., attenuation of muscle µ, Db10, and RTD impairments after the second bout).

### Magnitude and Time Course of Traditional EIMD Symptoms

As expected, the repetition of exercise bouts induced a RBE: symptoms of EIMD (CK, muscle soreness, MT, ROM) and NM alterations (MVC torque, VAL, RTD, twitch, Db10, Db100, Db10-to-Db100 Hz ratio) were less important, and recovered quicker after exercise 2. This is consistent with previous reports (Clarkson et al., 1992; Chen et al., 2012; Souron et al., 2018). The lesser decrease in VAL observed after the second exercise bout compared to the first bout suggests that the difference in MVC torque loss between the two exercises could be partly explained by neural factors, which is consistent with the literature. Indeed, Goodall et al. (2017) showed that the RBE may be partly explained by modifications in motor corticospinal drive (i.e., smaller reduction in VAL measured using motor cortical stimulation after the second bout). Less muscle damage experienced the days after the second bout could be associated with reduced nociceptive III and IV afferents activity and then decreased alterations at spinal and/or supra-spinal levels that could impact the VAL (Behrens et al., 2012). In line with Souron et al. (2018), we also found that peripheral (i.e., muscular) factors were largely implicated in the reduced NM function loss observed after the second bout. Indeed, the amplitudes of the evoked mechanical responses (Twitch, Db10 and Db100), and the Db10-to-Db100 Hz ratio were less affected after the second exercise compared to the first one. These results may suggest a proportional adaptation of contractile properties and/or E-C coupling (Gandevia, 2001), and more specifically, less impairments in calcium homeostasis. We showed that E-C coupling function estimated by the Db10-to-Db100 Hz ratio was more affected and for a longer duration after the first compared to the second exercise (48 vs. 4 h). This is consistent with Janecki et al. (2016) who found that low frequency fatigue was more affected 24 and 48 h after a first bout compared to a second bout of ECC of the elbow flexors.

### Magnitude and Time Course of Muscle µ

Consistent with the results reported in the literature (Lacourpaille et al., 2014), we found that resting VL and RF µ were significantly increased from POST to 24 h after the first exercise. One of the major hypothesis related to the increase in resting muscle µ after ECC is the perturbation of calcium homeostasis (i.e., increase in intramuscular calcium concentration) (Lacourpaille et al., 2014; Lacourpaille et al., 2017) induced by the disruption of structural muscle proteins (Paulsen et al., 2012). Indeed, the increase in intramuscular calcium concentration could trigger an augmentation of stable attached cross-bridges number (i.e., “contracture clots”) increasing muscle stiffness (Whitehead et al., 2001; Lacourpaille et al., 2014; Lacourpaille et al., 2017). However, the raise of RF µ exceed 48 h after the first exercise, when E-C coupling (i.e., Db10-to-Db100 Hz ratio) had fully recovered. These results suggest that the perturbation of calcium regulation was not the sole mechanism implicated in the increase of muscle µ. It could be that this late increase was due to the presence of edema (Howell et al., 1993). Indeed, even if we did not find an increase in thigh circumference, we found an increase of MT (peak: ∼+10%) up to 7 days for RF and at 24, 48 h and 7 days for VL, potentially reflecting muscle swelling (Clarkson et al., 1992). Based on these results, we cannot discard the effect of delayed fluid accumulation occurring after eccentric-biased exercise on muscle stiffness. It should be noted that ROM (i.e., the knee joint stiffness) was differently affected by the eccentric-biased exercise compared to resting µ (i.e., muscle stiffness) since ROM was only increased between 24 and 72 h. These discrepancies in terms of time-course could be explained by the fact that ROM represent a global joint stiffness whereas µ represent individual muscles.

### Long Term Elevation of Muscle µ as “Protective” Adaptation

RF µ remained elevated up to 14 days after the first exercise. These results are consistent with Lacourpaille et al. (2014) who reported an elevated resting µ 21 days after an ECC of the elbow flexors. These authors (Lacourpaille et al., 2014) suggested that muscles became stiffer in response to a damaging exercise bout to protect muscles against a subsequent EIMD. However, they were unable to confirm this hypothesis due to a lack of recovery of muscle function 21 days after the ECC, suggesting that the resting µ could be also due to persistent damage. In the present study, as the EIMD symptoms had disappeared (e.g., MVC torque loss) 14 days after the first exercise, it seems reasonable to assume that the RF µ increase at this time point could reflect muscle and/or connective tissue adaptations.

Furthermore, we showed that the increases in RF and VL µ observed 14 days after the first exercise (+28% and +10%, respectively) were correlated with the IP for magnitude of changes and IP of rate of recovery, suggesting a reduced elevation and quicker recovery of RF and VL µ after the second bout compared to the first bout. These correlations confirm that the muscle could became stiffer to confer protection against a subsequent damaging exercise bout, as suggested by Hyldahl et al. (2017). These authors reported that skeletal muscle extracellular matrix remodeling could increase passive stiffness the weeks following a bout of ECC. We also showed that the increase in RF µ observed 14 days after the first exercise was correlated with the functional protection conferred by the RBE (i.e., attenuation of Db10 and RTD impairments after the second bout). The fact that the long term increase in muscle stiffness conferred by a bout of eccentric-biased exercise mainly affected submaximal force production is consistent with the fact that these responses are more sensitive to changes in mechanical properties (Maffiuletti et al., 2016). From a functional point of view, changes in muscle stiffness could impact functional submaximal tasks that involve stretch shortening cycle such as walking or running (Saunders et al., 2004), where muscle stiffness is a key determinant of performance.

### Differences Between VL and RF Responses

We hypothesized that the magnitude of the alteration and the adaptive response would differ between the quadriceps muscles (i.e., RF vs VL) due to the fact that muscles are heterogeneously stretched during the exercise, owing to their differences in their anatomical (i.e., mono- and bi-articular muscles) properties (Green et al., 2012; Maeo et al., 2018). We showed that, despite RF and VL PPT being not differently affected after the exercises, RF MT was, on average, more increased than VL MT. Moreover, RF µ was more increased and for a longer duration compared to VL µ after the exercise 1 (up to 14 days and 24 h, respectively). These results are consistent with previous reports showing that RF is more affected than other muscle heads of the quadriceps after eccentric-biased exercise (Lacourpaille et al., 2017; Heales et al., 2018; Maeo et al., 2018; Xu et al., 2019; Ema et al., 2021).

RF µ increased 14 days after the first exercise (i.e., before the second exercise) whereas no increase in VL µ was observed at this time point. As RF µ was more affected than VL µ in terms of magnitude and time-course, it could be that the mechanical stress induced by the loaded DW was not sufficient to induce an important level of damage and a subsequent mechanical adaptation on the VL muscle. Indeed, Hyldahl et al. (2017) reported that the magnitude of the protective effect is related to the intensity of the initial bout. The present results are consistent with Ema et al. (2021) who found that the RBE was conferred on RF but not on VL after eccentric contractions of the knee extensors.

### Limitations

Given that fascicle orientation relative to ultrasound beam affects shear wave velocity (Eby et al., 2013), a difference in PA variation over time could differently affect the measurement between muscles (Lacourpaille et al., 2012). The increase in RF and VL PA observed between 4 and 48 h after the first exercise could decrease shear wave velocity, which could contribute to underestimate RF and VL µ at these time points. After exercise 2, no change in PA was observed for both RF and VL muscles. Therefore, PA should not affect changes in µ after the second exercise. As µ could be underestimated after the exercise 1 but not after the exercise 2, the magnitude of the difference in µ between the two exercise bouts could be underestimated. If this effect is present, it is certainly weak. Indeed, it should be noted that changes in PA are relatively small (<5°) in the present study, which should have a very small effect on shear wave velocity.

## Conclusion

The resting µ (i.e., muscle stiffness) increased to a smaller extent and for a shorter duration after a second bout of eccentric-biased exercise compared to the same bout performed 2 weeks earlier, representing the adaptations conferred by the RBE. The first damaging exercise induced a persistent (i.e., at least 2 weeks) elevation in muscle stiffness, potentially reflecting a mechanical adaptation involved in the RBE. Indeed, this persistent increase in muscle µ was associated with the functional protection conferred by the RBE. The mechanical adaptation is more evident on the muscle that has experienced the greatest changes in muscle stiffness after the first bout (i.e., RF). It could be that a certain level of mechanical stress is necessary to induce this persistent increase in muscle µ. More studies are needed to better understand the mechanisms involved in the modifications of muscle stiffness after eccentric-biased exercises. Muscle activation and muscle mechanical behaviors should be investigated during the exercise to better understand the differences in muscle µ changes between synergists muscles. Further studies should also investigate the mechanisms involved in the acute and long-term muscle mechanical properties modifications of muscle µ and their implication in the RBE.

### Clinical Application

The results of the present study suggest that SWE may be considered a valuable measurement tool, complementary to traditional EIMD markers, to assess protection of individual muscles conferred by the RBE (reduced alteration of the whole muscle system function). Moreover, the results provide a better understanding of the mechanical adaptations conferred by the RBE which could explain why muscles are more resistant after a first bout of eccentric exercise. It may have important applications in clinical and sport context to better adapt the exercise load. It could be interesting to modify the articular joints angles in order to adapt the recruitment of each muscle to modulate their mechanical stress and the potential mechanical adaptations, as previously tested by Ema et al. (2021).

## Data Availability

The original contributions presented in the study are included in the article/Supplementary Materials, further inquiries can be directed to the corresponding author.

## Abbreviations

CK, creatine kinase; Db10, doublet at 10 Hz; Db100, doublet at 100 Hz; DW, downhill walking; E-C, excitation-contraction; ECC, eccentric exercise; EIMD, exercise-induced muscle damage; IP, index of protection; MT, muscle thickness; NM, neuromuscular; PA, pennation angle; PPT, pain pressure threshold; RBE, repeated bout effect; RF, rectus femoris; ROI, region of interest; ROM, range of motion; RTD, rate of torque development; SWE, Shear-wave elastography; µ, shear elastic modulus; VAL, voluntary activation level; VL, vastus lateralis
