# Supplementary material for: Muscle Shear Elastic Modulus Provides an Indication of the Protection Conferred by the Repeated Bout Effect
Source: Front Physiol. 2022 Apr 29;13:877485. doi: 10.3389/fphys.2022.877485 (PMC9098813; doi:10.3389/fphys.2022.877485)
Supplement: Supplementary file 1 [file Table1.docx]

|  |  | PRE | | | POST | | | 4 h | | | 24 h | | | 48 h | | | 72 h | | | 168 h | | |
| --- | --- | --- | --- | --- | --- | --- | --- | --- | --- | --- | --- | --- | --- | --- | --- | --- | --- | --- | --- | --- | --- | --- |
| RF µ at 90° (kPa) | Exercise 1 | 9.7 | ± | 2.8 | 13.6 | ± | 3.7 | 15.5 | ± | 4.9 | 12.2 | ± | 4.0 | 11.1 | ± | 5.0 | 11.1 | ± | 3.5 | 10.5 | ± | 3.7 |
|  | Exercise 2 | 10.0 | ± | 2.4 | 11.2 | ± | 3.2 | 11.8 | ± | 2.6 | 10.3 | ± | 3.0 | 10.1 | ± | 3.0 | 9.6 | ± | 2.8 | 9.5 | ± | 2.4 |
| RF µ at 120° (kPa) | Exercise 1 | 23.0 | ± | 9.8 | 33.2 | ± | 13.0 | 34.7 | ± | 11.3 | 32.9 | ± | 14.3 | 27.9 | ± | 12.6 | 28.8 | ± | 15.9 | 26.3 | ± | 9.9 |
|  | Exercise 2 | 27.0 | ± | 10.3 | 31.4 | ± | 11.8 | 30.6 | ± | 9.8 | 25.9 | ± | 10.3 | 26.4 | ± | 11.8 | 24.7 | ± | 9.2 | 26.4 | ± | 12.3 |
| VL µ at 120° (kPa) | Exercise 1 | 8.3 | ± | 1.8 | 9.2 | ± | 1.7 | 9.2 | ± | 1.6 | 9.0 | ± | 1.7 | 8.7 | ± | 2.0 | 8.8 | ± | 1.9 | 8.6 | ± | 1.9 |
|  | Exercise 2 | 8.6 | ± | 1.9 | 9.5 | ± | 2.2 | 9.0 | ± | 1.9 | 8.8 | ± | 2.1 | 8.1 | ± | 1.4 | 8.1 | ± | 1.7 | 8.3 | ± | 2.0 |
| VL µ at 90° (kPa) | Exercise 1 | 13.6 | ± | 3.9 | 17.0 | ± | 5.4 | 16.1 | ± | 4.3 | 15.4 | ± | 3.9 | 14.7 | ± | 4.5 | 15.3 | ± | 5.4 | 13.7 | ± | 3.7 |
|  | Exercise 2 | 14.5 | ± | 4.4 | 16.4 | ± | 5.1 | 15.7 | ± | 4.3 | 15.1 | ± | 4.2 | 14.2 | ± | 3.8 | 13.2 | ± | 3.7 | 14.2 | ± | 5.1 |
